# Trends and determinants of infant and under-five childhood mortality in Vietnam, 1986–2011

**DOI:** 10.3402/gha.v9.29312

**Published:** 2016-02-29

**Authors:** Hwa-Young Lee, Dung Van Do, Sugy Choi, Oanh Thi Hoang Trinh, Kien Gia To

**Affiliations:** 1 JW Lee Center for Global Medicine, Seoul National University, College of Medicine, Seoul, Korea; 2 Faculty of Public Health, University of Medicine and Pharmacy at Ho Chi Minh city, Ho Chi Minh City, Vietnam

**Keywords:** infant mortality, under-five mortality, MICS, Vietnam, survey analysis, mortality trends

## Abstract

**Background:**

Although Vietnam has taken great efforts to reduce child mortality in recent years, a large number of children still die at early age. Only a few studies have been conducted to identify at-risk groups in order to provide baseline information for effective interventions.

**Objective:**

The study estimated the overall trends in infant mortality rate (IMR) and under-five mortality rate (U5MR) during 1986–2011 and identified demographic and socioeconomic determinants of child mortality.

**Design:**

Data from the Vietnam Multiple Indicator Cluster Surveys (MICSs) in 2000 (MICS2), 2006 (MICS3) and 2011 (MICS4) were analysed. The IMR and U5MR were calculated using the indirect method developed by William Brass. Unadjusted and adjusted odds ratios were estimated to assess the association between child death and demographic and socioeconomic variables. Region-stratified stepwise logistic regression was conducted to test the sensitivity of the results.

**Results:**

The IMR and U5MR significantly decreased for both male and female children between 1986 and 2010. Male children had higher IMR and U5MR compared with females in all 3 years. Women who were living in the Northern Midlands and Mountain areas were more likely to experience child deaths compared with women who were living in the Red River Delta. Women who were from minor ethnic groups, had low education, living in urban areas, and had multiple children were more likely to have experienced child deaths.

**Conclusion:**

Baby boys require more healthcare attention during the first year of their life. Comprehensive strategies are necessary for tackling child mortality problems in Vietnam. This study shows that child mortality is not just a problem of poverty but involves many other factors. Further studies are needed to investigate pathways underlying associations between demographic and socioeconomic conditions and childhood mortality.

## Introduction

**Fig. 1:**
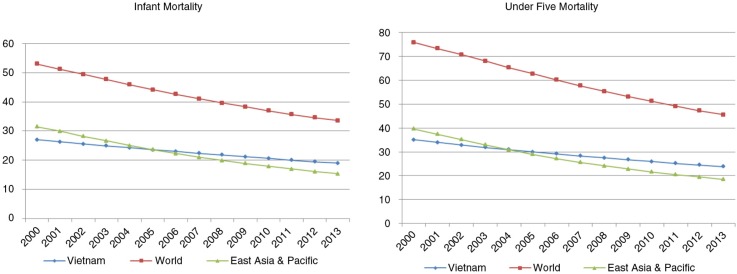
Trends in IMR and U5MR in Vietnam, regions and the world.

The under-five mortality rate (U5MR) and the infant mortality rate (IMR) have both fallen significantly in developing countries since the declaration of Millennium Development Goals (MDGs) in 2000. However, infant health remains a serious public health problem in Vietnam (1). In an effort to achieve MDG4 to reduce the child mortality, Vietnam reduced U5MR from 58 per 1,000 live births in 1990 to 24 per 1,000 in 2009 (2). In spite of this, since 2005, the IMR and U5MR in Vietnam have been higher than the average for countries in the East Asian and Pacific region. Currently, a large number of children in Vietnam still die before they reach their fifth birthday (see Fig. 1).^1^,^2^

In order to achieve significant reductions in childhood mortality in Vietnam, it is important to identify the vulnerable groups who have social characteristics strongly associated with childhood mortality so that appropriate action can be taken. However, to date, there have been limited epidemiological studies on this subject. Nga et al. identified the causes of neonatal death in Quang Ninh province and analysed this distribution by age at death, birth weight, and place of delivery from a clinical perspective (3). Pham et al. investigated the patterns of child mortality by gender (4). They found that the mortality rate for boys was much higher than for girls and that this was associated with parents’ educational levels, in particular those of the father. Knowles et al. performed a situational analysis of health inequity in Vietnam focussing on maternal and child mortality and showed that being an ethnic minority was the most important risk factor for high infant mortality (5). Hoa et al. examined the trends in neonatal, infant, and U5MR in a northern district of Vietnam between 1970 and 2000 and analysed socioeconomic differences in child survival over time (6). They identified dramatic reductions in IMR and U5MR during that period but not in the neonatal mortality rate (NMR). They also showed that U5MR and NMR were not associated with households’ economic situation although there was some association with mothers’ education and ethnicity. Swenson et al. investigated the factors influencing infant mortality with a specific focus on community characteristics and found that the most significant predictor of infant mortality was living area (7).

Likewise, previous studies in Vietnam were mostly based in specific regions, thus limiting the generalisability of their results. Others are outdated (3–7). Findings from previous studies have not been sufficient to identify the most vulnerable groups, or specify the types of interventions needed. This study aims to address the evidence gap in relation to taking action to reduce childhood mortality in Vietnam by 1) investigating overall trends in IMR and U5MR (1986–2011) by demographic and socioeconomic factors and 2) identifying the demographic and socioeconomic determinants of the early childhood deaths.

## Methods

### Data collection

Data from the Vietnam Multiple Indicator Cluster Surveys (MICSs) in 2000 (MICS2), 2006 (MICS3), and 2011 (MICS4) were used in this study. The Vietnam General Statistics Office has conducted the MICS every 5 years as a part of the global MICS programme. The United Nations Children's Fund (UNICEF) provided technical and financial support for all MICSs.

A stratified two-stage cluster sampling was designed to select eligible respondents. Six ecological regions were used as main strata. The primary sampling unit (PSU), a cluster for the survey, was defined on the basis of enumeration areas (EAs) and about 33 households out of 100 were selected in each EA acquiring a sampling probability of one-third. In each survey, there are three structured questionnaires, which are for households, 15–49 year-old women, and under-five year-old children. All the variables used for this study were derived from the women's datasets.

### Estimation of IMR and U5MR in Vietnam 1986–2010

To estimate IMR (the number of children dying at less than 1 year of age per 1,000 live births that year) and U5MR (the number of children dying at less than 5 years of age per 1,000 live births that year), the indirect method developed by William Brass in late 1960s (8) and modified by Coale and Trussel later (9) was used here. This method has been widely used in previous studies and provides consistent results with the direct method (10). A brief explanation is as follows:

To begin with, an average parity per woman *P*(*i*) and the proportion of dead children *D*(*i*) in each age group of women were estimated from the number of children ever born, the number of surviving children, and the number of women belonging to each age group. A multiplier *K*(*i*) was estimated for each age group of women [Eq(1)].

1 $$\text{K}(i)=\text{a}(i)+\frac{\text{p}(1)}{\text{p}(2)}+\text{b}(\text{i})\frac{\text{p}(2)}{\text{p}(3)}$$

Then, the probabilities of dying (*q*(*x*)) and the probabilities of surviving (*l*(*x*)) were determined as *q*(*x*) = *K*(*i*)*D*(*i*). The reference times were obtained using the model as below [Eq(2)].

2 $$\text{t}(\text{i})=\text{e}(\text{i})+\text{f}(\text{i})\frac{\text{p}(1)}{\text{p}(2)}+\frac{\text{p}(2)}{\text{p}(3)}$$

The *t*(*i*) values were then subtracted from the period of survey to produce the reference date. The *a*(*i*), *b*(*i*), *c*(*i*), *e*(*i*), *f*(*i*), and *g*(*i*) are constant coefficients and were taken from the North model life table (11), which was chosen because mortality patterns of the Vietnamese population are most similar to the North model life family, the one with low child and elderly mortality level (12, 13). Finally, the *q*(*x*) values from each group were transformed into _1_ *q*_0_ (infant mortality) and _5_ *q*_0_ (under-five mortality) for each reference point.

To identify trends of IMR and U5MR, each mortality rate was regressed on reference periods. Because the quadratic term was not significant in the curvature model, the final model was linear. The same procedures were applied to examine the trends in IMR and U5MR across the six regions, urban and rural living areas and maternal educational levels.

### Determinants associated with having a child death

Analyses were performed with MICS4 (2011) data because it provided the most recent data and the largest sample size.

The outcome variable is a child death experience reported by mothers. This was derived from answers to a question to the mother about whether she had ever given birth to a child who was born alive but later died.

Demographic and socioeconomic factors available in the MICS women's dataset that had been shown to be associated with child mortality in previous studies were the independent variables (14–17). The region was divided into six categories: Red River Delta; Northern Midlands and Mountain areas; Northern Central area and Central Coastal area; Central Highlands; South East and Mekong River Delta. Mothers’ ages were grouped into three categories; 15–24, 25–34, and 35–49 years. Living area was assessed by asking whether respondents were living in either urban or rural areas. The mothers’ educational level variable comprised three categories: ‘primary of less’ for respondents who reported that their highest level of education was grade five, ‘secondary’ for respondents who reported that their highest level of education was between grade six and twelve, and ‘tertiary’ for respondents who reported that they finished professional school, college or university and above. The household wealth index was used as a proxy for economic status. Wealth scores were derived by Principal Component Analysis using information collected on each household's ownership of consumer goods and amenities in related to household wealth. The scores were divided into quintiles from the poorest to the wealthiest. Mothers’ ethnicity was classified as one of the two groups: Kinh, which is the main ethnic group accounting for about 84% of Vietnamese people, and non-Kinh. Marital status was grouped into two categories: ‘married’ meaning ‘living with a spouse’ and ‘not married’ which included ‘never married’ and ‘widowed/divorced/separated’. It was assumed that the number of times a woman had given birth could be a confounder and therefore the number of children ever born to a mother was also included as a continuous independent variable.

The unit of analysis in this study was the mother. Univariable and multivariable logistic regressions were performed producing unadjusted and adjusted odds ratios (ORs) for each independent variable. We also performed region-stratified, multivariable stepwise logistic regression as a sensitivity analysis. Here, mothers’ education levels were dichotomised as lower level (finished junior high school or less) and higher level (finished senior high school or above). All analyses were carried out using R statistical software (18).

## Results

### Estimates of IMR and U5MR in Vietnam during 1986–2010

Weighted and non-weighted numbers of women and children ever born, alive, and dead are shown in Table 1. The age distribution of women in this study was roughly consistent with the population pyramid except that the number of women aged 40–44 in MICS3 was larger than the age groups 30–34 and 25–30 years. The number of children ever born and dead was the lowest for women aged 15–19 and 20–24 years. The average number of children ever born decreased consistently across the three time points.

**Table 1:** Number of women and children ever born, alive and dead in each age group in Vietnam, 2001, 2006, and 2011

Survey	Age group	FP(i)	CEB(i)	CA(i)	CD(i)	wFP(i)	wCEB(i)	wCA(i)	wCD(i)
MICS2	15–19	1,900	71	68	3				
	20–24	1,398	823	788	35				
	25–30	1,341	2,143	2,062	81				
	30–34	1,337	3,156	3,008	148				
	35–39	1,313	4,016	3,765	251				
	40–44	1,181	4,195	3,889	306				
	45–49	782	3,166	2,899	267				
MICS3	15–19	1,839	59	59	0	1768.3	48.3	48.3	0.0
	20–24	1,391	761	752	9	1358.7	692.8	685.7	7.1
	25–30	1,195	1,671	1,631	40	1168.0	1497.0	1465.4	31.6
	30–34	1,218	2,556	2,474	82	1207.4	2409.5	2333.0	76.5
	35–39	1,273	3,182	3,056	126	1306.0	3162.2	3041.4	120.8
	40–44	1,345	4,064	3,879	185	1385.1	3981.4	3803.9	177.5
	45–49	1,210	4,154	3,846	308	1276.4	4214.6	3899.3	315.3
MICS4	15–19	1,760	88	87	1	1697.6	82.0	80.6	1.5
	20–24	1,632	805	792	13	1609.1	818.6	806.4	12.2
	25–30	1,790	2,222	2,177	45	1810.4	2213.4	2175.1	38.3
	30–34	1,738	3,197	3,147	50	1810.6	3308.5	3258.1	50.4
	35–39	1,642	3,629	3,509	120	1661.6	3655.9	3529.0	127.0
	40–44	1,653	4,134	3,931	203	1620.8	3962.8	3781.5	181.3
	45–49	1,448	4,052	3,801	251	1452.9	4033.5	3796.6	236.8

FP(i): Female population; CEB(i): Children ever born; CA(i): Children alive; CD(i): Children dead; wFP(i): weighted Female population; CEB(i): weighted Children ever born; CA(i): weighted Children alive; CD(i): weighted Children dead.

### Trends of IMR and U5MR in Vietnam during 1986–2010

Table 2 provides estimates of IMR and U5MR based on the data from Table 1.

**Table 2:** IMR and U5MR in Vietnam, 2001, 2006, and 2011

Survey	Age group (years)	X	Weight	q_x_	Time Ref	Ref.P	IMR	U5MR
MICS2	15–19	1	0	0.0529	0.7150	1999.7	0.0529	0.0794
	20–24	2	0.2	0.0478	1.7687	1998.6	0.0415	0.0594
	25–30	3	1.2	0.0391	3.4962	1996.9	0.0324	0.0434
	30–34	5	1.2	0.0483	5.6867	1994.7	0.0351	0.0483
	35–39	10	1.2	0.0651	8.1895	1992.2	0.0402	0.0572
	40–44	15	0.8	0.0750	10.9471	1989.5	0.0423	0.0609
	45–49	20	0.4	0.0861	13.9964	1986.4	0.0434	0.0628
MICS3	15–19	1	0	0.0000	0.6008	2006.1		
	20–24	2	0.2	0.0115	1.7222	2005.0	0.0110	0.0126
	25–30	3	1.2	0.0215	3.6153	2003.1	0.0192	0.0232
	30–34	5	1.2	0.0322	6.0184	2000.7	0.0256	0.0322
	35–39	10	1.2	0.0392	8.7503	1998.0	0.0277	0.0352
	40–44	15	0.8	0.0451	11.6746	1995.0	0.0290	0.0373
	45–49	20	0.4	0.0752	14.7279	1992.0	0.0388	0.0548
MICS4	15–19	1	0	0.0218	0.7951	2010.2	0.0218	0.0268
	20–24	2	0.2	0.0163	1.9584	2009.0	0.0154	0.0182
	25–30	3	1.2	0.0176	3.8103	2007.2	0.0159	0.0188
	30–34	5	1.2	0.0155	6.1077	2004.9	0.0133	0.0155
	35–39	10	1.2	0.0358	8.6964	2002.3	0.0256	0.0322
	40–44	15	0.8	0.0465	11.4933	1999.5	0.0296	0.0385
	45–49	20	0.4	0.0593	14.5037	1996.5	0.0322	0.0432

q_x_: probability of children mortality at x years old in specific age group of women; Reference period (Ref.P): year where the mortality rates are applied; Time reference (Time Ref) is the distance between time of conduction survey and reference period.

**Fig. 2:**
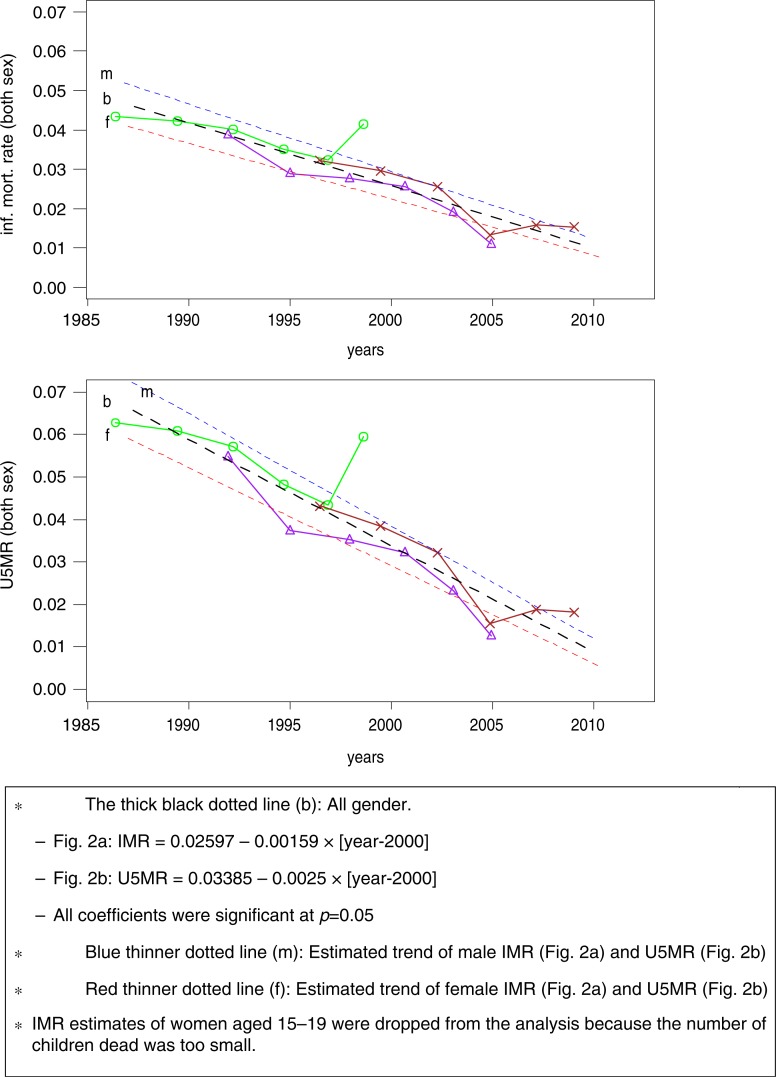
Estimated trends in (a) IMR and (b) U5MR from 1986 to 2010 in Vietnam derived from MICS2 (green line with marked ○), MICS3 (purple line with marked Δ), and MICS4 (brown line with marked ×).

The results of linear regressions are shown in Fig. 2a and b. Regression lines for IMR and U5MR indicate that the number of dead children under age 1 and 5 per 1,000 decreased respectively by 1.6 and 2.5 on average per year. This declining trend occurred among both male and female children. The IMR and U5MR for male children were consistently higher than those for female children between 1986 and 2010.

**Fig. 3:**
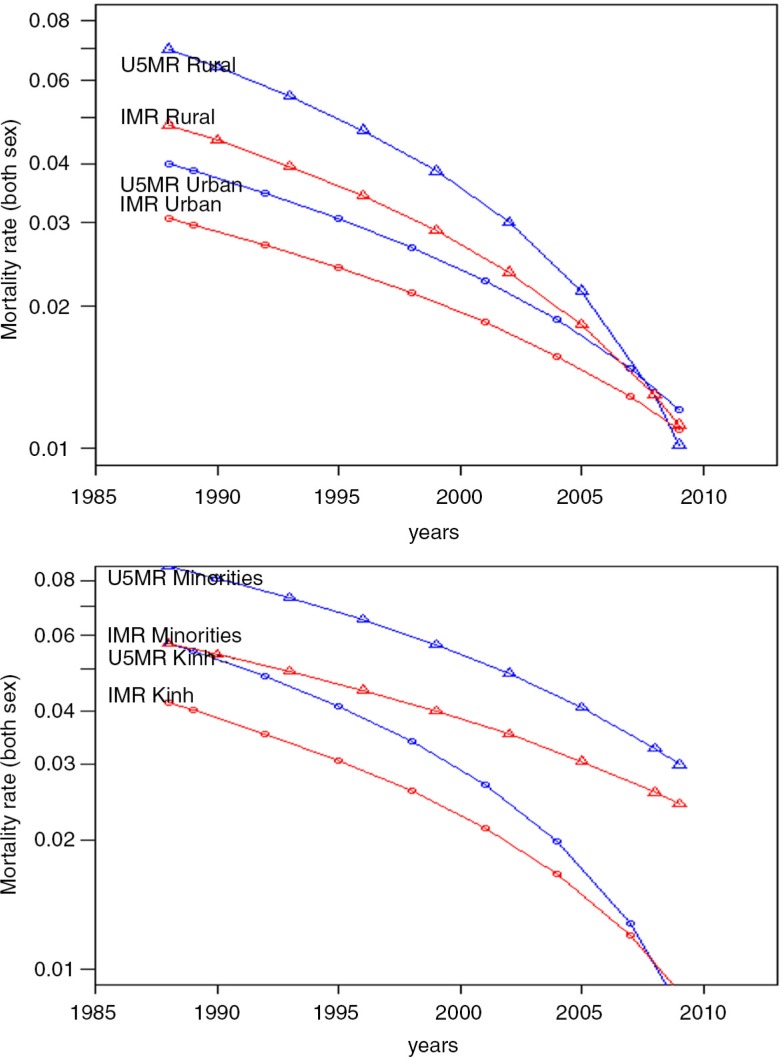
Trends in IMR (red line) and U5MR (blue line) (a) in urban areas (○) and rural areas (Δ) from 1986 to 2010 in Vietnam and (b) in Kinh group (○) and Ethnic Minorities (Δ) from 1986 to 2010 in Vietnam.

3 presents estimates of the IMR and U5MR in 2000 and regression coefficients by region, living area and mothers’ educational level. Overall, the IMR and U5MR decreased. The large gap in the IMR and U5MR between urban and rural areas (IMR: 0.03 versus 0.05, U5MR: 0.04 versus 0.07) in 1987 narrowed to zero around 2010 (Fig. 3a). However, this was not the case for the gap between the minor and major ethnic groups. Although the IMR and U5MR decreased for both ethnic groups during the same period, the reduction in the Kinh ethnic group was larger, leading to a wider gap between two groups around 2010 (Fig. 3b).

**Table 3:** Estimated IMR and U5MR in 2000 and the slopes by region, living area, and education of mother in Vietnam, 1986–2000

	IMR at 2000	IMR slope	U5MR at 2000	U5MR slope
Region				
Red River Delta	0.0253	−0.0009	0.0332	−0.0014
Northern Midlands and Mountain areas	0.0298	−0.0012	0.0400	−0.0020
Northern Central area and Central Coastal area	0.0275	−0.0018	0.0365	−0.0029
Central Highlands	0.0275	−0.0017	0.0365	−0.0028
South East	0.0180	−0.0010	0.0220	−0.0014
Mekong River Delta	0.0256	−0.0023	0.0341	−0.0037
Living area				
Urban	0.0194	−0. 0009	0.0240	−0.0013
Rural	0.0271	−0.0018	0.0357	−0.0028
Mother's education				
Primary	0.0347	−0.0022	0.0476	−0.0038
Secondary	0.0296	−0.0018	0.0396	−0.0030
Tertiary	0.0205	−0.0014	0.0258	−0.0020

### Determinants of child mortality

The total number of women aged 15–49 in MICS4 was 12,115, of which 8,179 women had ever given birth to a child. Table 4 presents an overview of child deaths by maternal demographic and socioeconomic characteristics.

**Table 4:** Distribution of child deaths by demographic and socioeconomic characteristics in Vietnam, 2011

Categories	*N*	Child death (%)
Region		
Red River Delta	1,239	54 (4)
Northern Midlands and Mountain areas	1,503	142 (9)
Northern Central area and Central Coastal area	1,273	96 (8)
Central Highlands	1,467	117 (8)
South East	1,335	57 (4)
Mekong River Delta	1,362	86 (6)
Mother's age (years)		
15–24	702	13 (2)
25–34	2,765	76 (3)
35–49	4,712	463 (10)
Living area		
Urban	3,439	182 (5)
Rural	4,740	370 (8)
Mother's education		
Primary or lower	2,181	221 (10)
Secondary	4,817	304 (6)
Tertiary	1,181	27 (2)
Wealth index quintiles		
Poorest	1,630	182 (11)
Second	1,393	123 (9)
Middle	1,529	95 (6)
Fourth	1,729	82 (5)
Richest	1,898	69 (4)
Ethnicity		
Kinh	6,860	380 (6)
Non-Kinh	1,319	172 (13)
Marital status		
Currently married	7,430	498 (7)
Not married	749	54 (7)
Total	8,179	552 (7)

Overall, the percentage of women who had experienced a child death was less than 10% in most of the categories. However, there were some gradients across categories within variables. For example, of the women who were older, had lower educational levels and lower wealth, higher proportions experienced child mortality.

Table 5 shows the association between child deaths and maternal demographic and socioeconomic factors. With the exception of marital status, all the variables showed a significant association with child death in the unadjusted regression, but this attenuated in the adjusted regression.

**Table 5:** Unadjusted and adjusted logistic regression for child deaths in Vietnam, 2011

	Unadjusted		Adjusted	
		
	OR	CI	OR	CI
Region				
Red River Delta				
Northern Midlands and Mountain areas	2.29^a^	1.66–3.16	1.58^a^	1.08–2.32
North Central area and Central Coastal area	1.79^a^	1.27–2.52	1.17	0.81–1.69
Central Highlands	1.90^a^	1.36–2.65	0.81	0.55–1.17
South East	0.98^b^	0.67–1.43	0.99	0.66–1.49
Mekong River Delta	1.48^a^	1.04–2.10	1.30	0.88–1.92
Mother's age (years)				
15–24				
25–34	1.50	0.83–2.71	0.89	0.49–1.64
35–49	5.78^a^	3.31–10.08	1.66	0.92–2.98
Number of children	2.44^a^	2.28–2.61	2.36^a^	2.17–2.56
Living area				
Urban				
Rural	1.52^a^	1.26–1.82	0.78^b^	0.61–0.98
Mother's education				
Primary or lower				
Secondary	0.60^a^	0.50–0.72	1.52^a^	1.18–1.95
Tertiary	0.21^a^	0.14–0.31	1.05	0.64–1.72
Wealth index				
Poorest				
Second	0.77^b^	0.60–0.97	1.12	0.82–1.51
Middle	0.52^a^	0.40–0.68	0.95	0.68–1.34
Fourth	0.39^a^	0.30–0.52	0.86	0.60–1.25
Richest	0.30^a^	0.22–0.40	0.74	0.49–1.14
Ethnicity				
Kinh				
Non-Kinh	2.56^a^	2.11–3.09	1.57^a^	1.15–2.14
Marital status				
Currently married				
Not married	1.08	0.81–1.45	0.92	0.75–1.13

a *p*<0.01

b *p*<0.05.

In multivariable logistic regression, mothers from the Northern Midlands and Mountain areas were more likely to experience child deaths compared with those from the Red River Delta (OR: 1.58, 95% confidence interval [CI] = 1.08–2.32). The number of children ever born to a mother was also significantly associated with the likelihood of a child death. However, the odds of experiencing a child death reduced from 2.44 (95% CI = 2.28–2.61) in the unadjusted regression to 2.36 (95% CI = 2.17–2.56) in the adjusted regression. [Table T0006]

**Table 6 T0006:** Results of region-stratified stepwise logistic regression for child deaths in Vietnam in 2011

	Red River Delta	Northern Midlands and Mountain areas	North Central area and Central Coastal area	Central Highlands	South East	Mekong River Delta
(Intercept)	0.0011^a^	0.0099^a^	0.0054^a^	0.0062^a^	0.0022^a^	0.0067^a^
No children ever born	4.08^a^	3.08^a^	2.46^a^	1.99^a^	2.43^a^	2.43^a^
Low education (mothers)		0.58^c^		2.03^d^	2.64^c^	
Minority ethnic	11.03^b^		4.17^a^	1.54^d^		
Rural resident		0.61^c^		0.59^c^		

a *p*<0.001

b *p<*0.01

c *p<*0.05

d *p<*0.1.

Women who were living in rural areas were more likely to have experienced a child death compared with those living in urban areas (OR: 1.52, 95% CI = 1.26–1.82) in the univariable analysis. However, this association was reversed when adjusting for covariates (OR: 0.78, 95%CI=0.61–0.98). The relationship between mothers’ education level and child death in the univariable regression also reversed when covariates were added. In the multivariable analysis, women who completed secondary school were more likely to experience a child death compared with those who attained primary or lower education (OR: 1.52, 95%CI: 1.18–1.95). In the multivariable analysis, the odds of experiencing a child death was higher for women in minor ethnic groups compared with Kinh women (OR: 1.57, 95%CI: 1.15–2.14).

The direction of the coefficients for the predictor variables across the six regions in the sensitivity analyses remained the same as the main analyses except for education (Table 6). Although low education was associated with higher odds of experiencing a child death in the Central Highlands and South East, it was associated with lower odds of a child death in the Northern Midlands and Mountain areas.

## Discussion

The present study estimated the IMR and U5MR and their trends in Vietnam over the period 1986–2010 and investigated demographic and socioeconomic factors associated with child mortality. There are some noteworthy findings.

First, boys showed higher IMRs and U5MRs compared with girls in all years. This is consistent with the research by Hoa et al. who analysed socioeconomic differences in child survival during 1970–2000 (6). They found that boys had higher mortality risks than girls because boys are more vulnerable during their neonatal period, particularly in the case of preterm birth. According to Ingemarsson, preterm baby boys have double risks of dying during their first year compared to baby girls (19). Our finding suggests that antenatal and neonatal care have to be strengthened to improve child mortality and in particular for baby boys.

Second, the falling rates for both IMR and U5MR were much higher in rural areas. This can be explained by the recent reductions in rural poverty rates in Vietnam. For example, the poverty rate in rural areas fell substantially from 66.4% in 1993 to 18.7% in 2008 (20). Although the poverty rate in urban areas also declined during the same period, urban poverty is a relatively recent phenomenon with many urban poor living in recently urbanised peripheral districts in cities. These people are mostly low-income migrants who also suffer from various social problems. As a result, there have been limited opportunities for further reductions of child mortality in urban areas since 2008 (20).

The third intriguing finding is that although the mortality rate decreased significantly in both ethnic groups, the trend in the ethnic minorities was less favourable than the Kinh ethnic group, as seen in Fig. 3b. It is well recognised in Vietnam that ethnic minorities usually lack physical and social assets, and reside in remote areas with limited access to various governmental subsidy programmes and welfare benefits (21, 22). Another possible reason for the slow reduction in child mortality among ethnic minorities has to do with traditional practices including home deliveries, rituals surrounding birth and negative perceptions of healthcare personnel (23). Language barriers can also be a problem, discouraging ethnic minorities from seeking formal healthcare services. As such, slower progress in reducing child mortality rates for ethnic minorities in Vietnam is not only due to poverty but to other social factors.

The results of the analysis, which was intended to identify the determinants of child deaths, also provide a few notable findings. First, compared with women living in the Red River Delta, women in the Northern Midlands and Mountain areas had higher odds of experiencing a child death. The Northern Midlands and Mountain areas have a relatively hot and humid subtropical climate throughout the year. About six to ten hurricanes and tropical depressions causing floods occur annually in those areas. These conditions directly threaten lives, especially the vulnerable, including young children in the region. In addition, mountainous or half-mountainous geographical features may hamper access to healthcare services. Poor and harsh climatic conditions and low accessibility to healthcare services may therefore help explain the higher odds of child mortality in the region.

Unlike some previous studies, those living in rural areas had lower odds of experiencing a child death (22). A plausible explanation for this can be found in emergence of urban poverty, as already mentioned. A mini-crisis in early 2008 in Vietnam had a negative impact on the poor, who were mainly living in marginalised urban areas. In addition, they also suffer from various non-income-related aspects of poverty, such as the hazards of pollution, risks to personal safety, and harsh work and housing conditions (20). These are possible reasons why women living in rural areas were less likely to experience a child death compared with women in urban areas.

A final finding is the positive association between maternal education and child death, which also differs from the results of previous studies (17, 24). However, the region-stratified sensitivity analyses provided different outcomes. The relationship between maternal education and a child death was statistically significant only in the two regions (Northern Midlands and Mountain areas and Central Highlands). However, the association was negative for women only in the North Central area and Central Coastal area. Though we tried adding the independent variables stepwise, starting with education in the Northern Midlands and Mountain areas dataset, the direction of the coefficient changed from positive to negative when we added the number of children (results not shown). This suggests that mothers with higher education tended to have a fewer children. That is, the number of children acted as a confounding factor, which accords with the results of a study by Adebowale et al. (22). We suggest that the reasons for the higher likelihood of child death among mothers with higher education in the Northern Midlands and Mountain areas should be explored in future studies.

### Strengths and limitations

To our knowledge, this is the first study of its kind to investigate recent child mortality trends in Vietnam using the indirect method. Unlike previous studies, we controlled for the effect of the number of children. Yet, this study has also some limitations to consider.

Although there is evidence that occupation, religion, and living environment of mothers are risk factors for child deaths (25, 26), these variables were not available in our dataset. Additionally, experiencing child mortality is likely to be associated with community-level factors such as public sanitation facilities, birth delivery practices, and perceptions about traditional medicines (27). Additional analysis using multilevel techniques may provide further insights into child mortality in Vietnam. We also acknowledge that the survey may have underestimated child deaths because of the sensitivity of the topic for mothers. However, we are unable to assert whether and to what extent this may have occurred.

## Conclusions

This study provides evidence and guidance for future policy to address childhood mortality in Vietnam. Interventions should focus on the most vulnerable groups such as the newly emerging urban poor and also ethnic minorities. These policies should not only involve socioeconomic improvements but should also include interventions for cultural and behavioural change. There is a need for further studies to investigate pathways underlying the associations between demographic and socioeconomic conditions and childhood mortality in Vietnam.

## Footnotes

1 http://data.worldbank.org/indicator/SP.DYN.IMRT.IN

2 http://data.worldbank.org/indicator/SH.DYN.MORT
